# Strained Ammonium Precursors for Radiofluorinations

**DOI:** 10.1002/open.202200039

**Published:** 2022-06-23

**Authors:** Falco Reissig, Constantin Mamat

**Affiliations:** ^1^ Institut für Radiopharmazeutische Krebsforschung Helmholtz-Zentrum Dresden-Rossendorf Bautzner Landstraße 400 01328 Dresden Germany; ^2^ Fakultät Chemie und Lebensmittelchemie Technische Universität Dresden 01062 Dresden Germany

**Keywords:** azetidinium, aziridines, fluorination, ring opening, strained rings

## Abstract

The increasing application of positron emission tomography (PET) in nuclear medicine has stimulated the extensive development of a multitude of novel and versatile techniques to introduce fluorine‐18, especially for the radiolabelling of biologically or pharmacologically active molecules. Taking into consideration that the introduction of fluorine‐18 (t_1/2_=109.8 min) mostly proceeds under harsh conditions, radiolabelling of such molecules represents a challenge and is of enormous interest. Ideally, it should proceed in a regioselective manner under mild physiological conditions, in an acceptable time span, with high yields and high specific activities. Special attention has been drawn to 2‐fluoroethyl and 3‐fluoropropyl groups, which are often the active sites of radiofluorinated compounds. Precursors containing an ammonium leaving group – such as a strained azetidinium or aziridinium moiety – can help to overcome these obstacles leading to a convenient and mild introduction of [^18^F]fluoride with high radiochemical yields.

## Graphical abstract

**Fluorine‐18 is the key to open the chain**. This review covers the convenient procedure for a radiofluorination of aziridines, aziridinium salts or azetidinium salts. These three‐ and four‐membered saturated heterocyclic ring systems were easily opened by the nucleophilic attack of [^18^F]fluoride to yield either 2‐[^18^F]fluoroethyl or 3‐[^18^F]fluoropropyl moieties. Since these groups are present in a variety of (radio)fluorinated compounds, these reactions are of (radio)pharmaceutical interest.

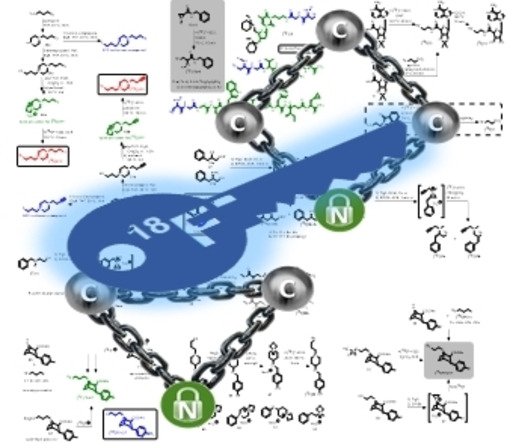

## 1 Introduction

The positron emitter fluorine‐18 is the most and widely used radionuclide for the production of radiopharmaceuticals for positron emission tomography (PET) apart from further short‐lived nonmetallic radionuclides carbon‐11, nitrogen‐13, oxygen‐15,[^1^, ^2^, ^3^] and iodine‐124[^4^, ^5^] as well as, for example, the radiometals gallium‐68, copper‐64, scandium‐43/‐44 and zirconium‐89.^[6]^ Special attention has to be paid to the method of the [^18^F]fluoride introduction. Commonly known radiolabelling methods like nucleophilic and electrophilic substitutions, metal catalyst mediated mechanisms, ligand exchange or prosthetic groups are widely used to regioselectively achieve high radiochemical yields.[^7^, ^8^] Normally, the introduction of fluorine‐18 proceeds via two main routes: the electrophilic way is based on the use of [^18^F]F_2_,^[9]^ mostly with low molar activities. In contrast, [^18^F]fluoride is used^[10]^ for the nucleophilic way, typically with high molar activities of the resulting radiotracer. Usually, no‐carrier‐added [^18^F]fluoride is applied for nucleophilic reactions with high molar activity in form of its K[^18^F]F‐K222 (Kryptofix) complex, proceeding as s_n_ 2‐type substitutions for aliphatic moieties and as S_N_Ar‐type substitutions for (hetero)aromatic moieties.^[11]^ Appropriate leaving groups such as halogens or sulfonates are required for the introduction into aliphatic groups, combined with almost water‐free conditions, a sufficient solubility in organic solvents and high temperatures, since fluoride is a bad nucleophile.^[12]^ In the case of aromatic nucleophilic substitution, leaving groups like F (isotope exchange), Me_3_N^+^, NO_2_ or other halogens have been used.^[13]^

**Scheme 1:**
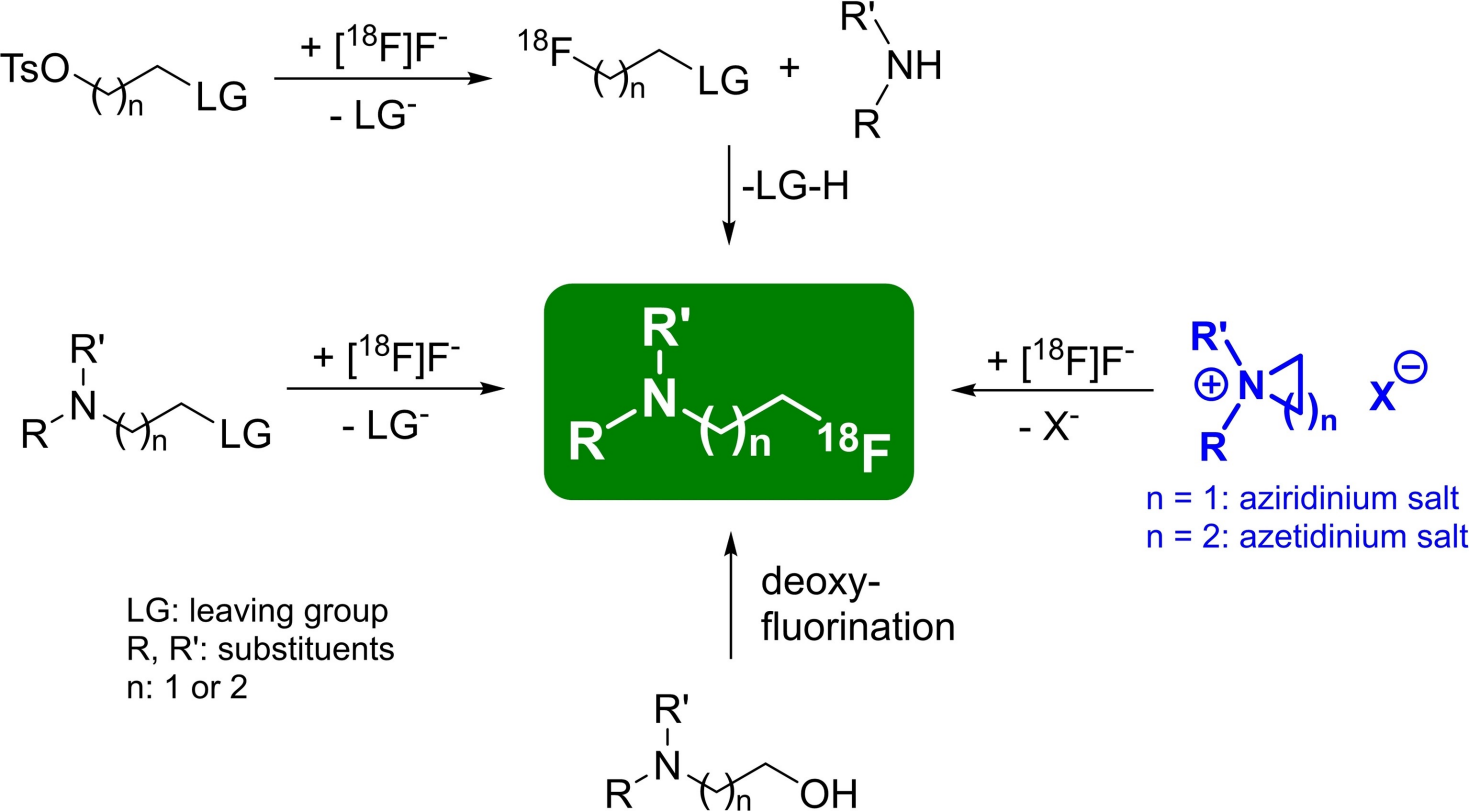
Four different general possibilities to obtain radiotracers containing a [^18^F]fluoropropyl or [^18^F]fluoroethyl moiety.

Tertiary 2‐[^18^F]fluoroethylamine and 3‐[^18^F]fluoropropylamine moieties are structural elements commonly found in several ^18^F‐radiotracers. The four most commonly used and well‐established approaches for the preparation of such radiotracers are illustrated in Scheme 1. The first approach comprises the preparation of a [^18^F]fluoropropyl or ‐ethyl moiety (e. g., mostly 1‐bromo‐3‐[^18^F]fluoropropane or 2‐[^18^F]fluoroethyl tosylate),^[14]^ followed by subsequent introduction of this ^18^F‐building block into secondary amine precursors using a nucleophilic displacement reaction. Overall radiochemical yields (RCYs) ranging from 10 % to 51 % were obtained using this two‐step reaction.[^15^, ^16^, ^17^, ^18^, ^19^, ^20^] In the second approach, a tertiary amine with an ethyl or propyl residue, equipped with a leaving group at the terminal carbon atom, is directly reacted with [^18^F]fluoride. Using tosylate or mesylate as leaving groups led to RCYs ranging from 33 % to 70 %,[^15^, ^21^, ^22^, ^23^, ^24^, ^25^, ^26^, ^27^] whereas a RCY of 88 % was reported when bromine was employed as the leaving group.^[28]^ A third approach centres around deoxy(radio)‐fluorinations which are also known for the introduction of fluorine‐18 via a direct replacement of OH groups using, for example, ^18^F‐versions of DAST (diethylaminosulfur trifluoride), deoxo‐fluor (bis(2‐methoxyethyl)aminosulfur trifluoride) or PyFluor (2‐pyridinesulfonyl fluoride).[^29^, ^30^, ^31^, ^32^]

The fourth approach consists of the application of strained cyclic ammonium salts or small cyclic amines (3‐/4‐membered rings) as precursors. The reactions of nucleophiles with aziridines or aziridinium ions (3‐membered rings), causing the ring opening of the substituted heterocycles, are reported to be facile,^[33]^ especially for the introduction of fluorine‐18. However, only little is known about ring‐opening reactions of azetidinium salts (4‐membered rings). These reactions have been reported to lead to unbranched propyl chains when the heterocycle is unsubstituted.[^34^, ^35^, ^36^, ^37^, ^38^]

Derivatives of α‐ and β‐amino acids bearing a fluorine atom at the vicinal aliphatic position have gained widespread applications, for instance in peptide/protein chemistry combined with protein recognition. They represent an important class of enzyme inhibitors, antitumor and antibacterial agents, samples for ^19^F NMR analyses and radiotracers for PET when radiolabelled with fluorine‐18.[^39^, ^40^, ^41^]

## 2 Aziridines and Aziridinium Salts

**Scheme 2:**
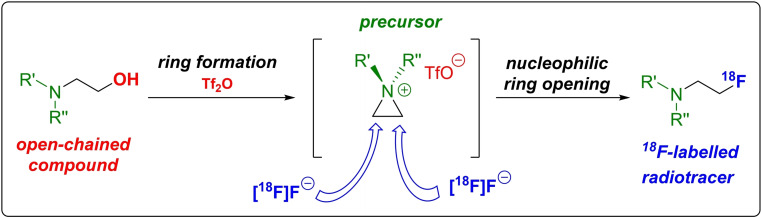
Procedure for the formation of the aziridinium salt precursor using the triflate route and subsequent ^18^F‐introduction by nucleophilic ring opening.

Aziridines and aziridinium salts are three‐membered heterocyclic compounds with one nitrogen atom in the ring, which, in the case of aziridinium salts, is positively charged. A nucleophilic attack is possible at both carbon atoms of the ring systems, leading to an opening of the strained three‐membered ring[^42^, ^43^] and, in the case of using fluoride as nucleophile, to the formation of a 2‐fluoroethyl moiety (Scheme 2).

**Scheme 3:**
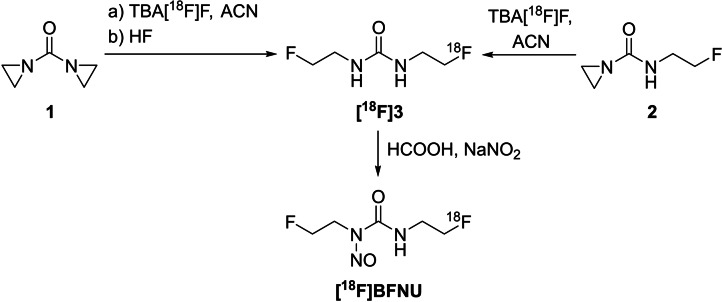
Synthesis of nitrosourea derivative **[^18^F]BFNU** in a two‐step preparation procedure.

Aziridines were first used for the synthesis of ^18^F‐labelled 2‐fluoroethyl‐nitrosourea derivatives.^[44]^ These compounds were reported for the treatment of patients with malignant brain tumours, including brain gliomas.[^45^, ^46^] 1,1’‐Carbonylbisaziridine (**1**) or *N*‐(2‐fluoroethyl)aziridine‐1‐carboxamide (**2**) were used for the preparation of **[^18^F]BFNU** (Scheme 3). The radiolabelling was performed in acetonitrile at 145 °C for 20 min in the presence of 18‐crown‐6. In the case of using the diaziridine precursor **1**, HF was added afterwards to enforce the ring opening of the second aziridine. The nitration of **[^18^F]3** in the last step was conducted in formic acid as solvent with sodium nitrite at 0 °C for 5 min, followed by a purification via HPLC with RCYs ranging from 5 % to 10 % for **[^18^F]BFNU**.

**Scheme 4:**
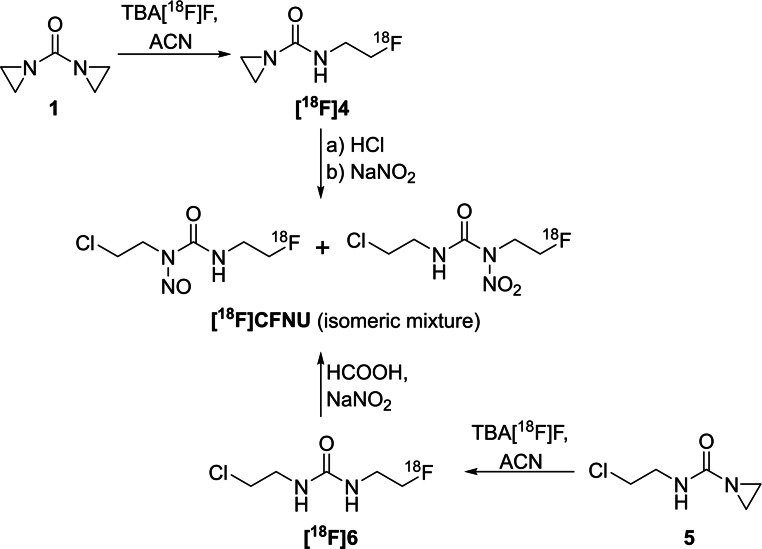
Synthesis of **[^18^F]CFNU** as mixture of regioisomers in a two‐step procedure starting either from precursor **1** or **5**.

Additionally, the second radiotracer **[^18^F]CFNU** was obtained as mixture of regioisomers after radiolabelling (Scheme 4) with RCYs ranging from 8 % to 15 %. Here, either 1,1’‐carbonylbisaziridine (**1**) or *N*‐(2‐chloroethyl)aziridine‐1‐carboxamide (**5**) were used as starting materials. The radiofluorination was performed under the above‐mentioned conditions also used for **[^18^F]BFNU**. For the diaziridine precursor **1**, HCl was used to ring‐open the second aziridine moiety in the second step after labelling. The last step of both routes involved the nitration of **[^18^F]4** and **[^18^F]6**, respectively, as described above, leading to an isomeric mixture of **[^18^F]CFNU** after HPLC purification.

**Scheme 5:**
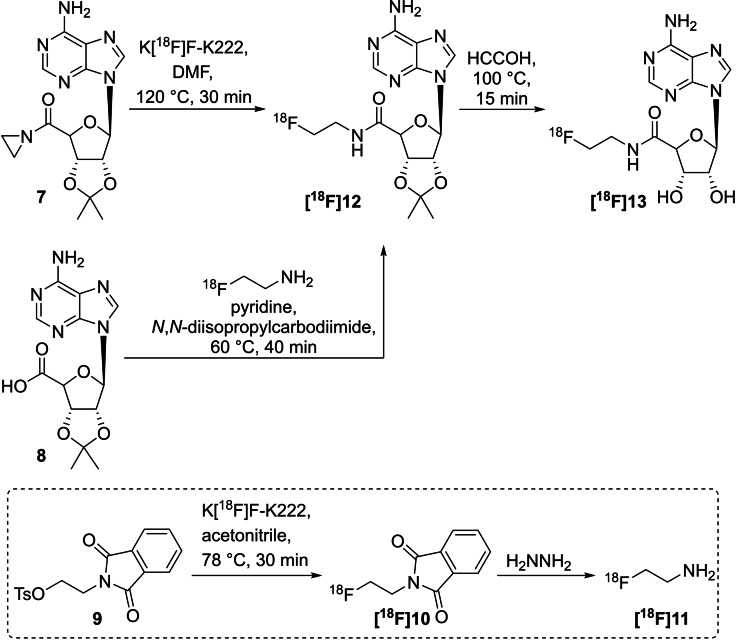
Two radiolabelling procedures to prepare the isopropyl‐protected radiotracer **[^18^F]12** followed by deprotection to **[^18^F]13** starting either from aziridine **7** or alternatively from **8** in a two‐step synthesis procedure with 2‐[^18^F]fluoroethylamine **[^18^F]11** (dashed box).

In 2000, Trón and colleagues synthesised ^18^F‐labelled adenosine **[^18^F]13** from the isopropylidene protected aziridine precursor **7** in two steps (Scheme 5).^[47]^ The precursor **7** was obtained from uronic acid **8** which was reacted with aziridine and *N,N*‐diisopropylcarbodiimide as the coupling agent. The nonradioactive reference compound was prepared from uronic acid **8** and 2‐fluoroethylamine. The radiolabelling was accomplished with aziridine precursor **7**, which was treated with K[^18^F]F‐K222 in DMF at 120 °C for 30 min. Unfortunately, isopropyl‐protected 2‐[^18^F]fluoroethyl‐adenosine **[^18^F]12** was obtained in only 1.1 % RCY. Based on this, an alternative route using 2‐[^18^F]fluoroethylamine (**[^18^F]11**) as radiolabelling building block was used, leading to the isolation of isopropyl‐protected compound **[^18^F]12** in a high RCY of 94±13 % from 2‐[^18^F]fluoroethylamine (**[^18^F]11**). The deprotection of **[^18^F]12** to obtain the final radiotracer **[^18^F]13** was achieved under acidic conditions and in quantitative yields. The radiolabelling procedure as well as the alternative way are shown in Scheme 5.

**Scheme 6:**
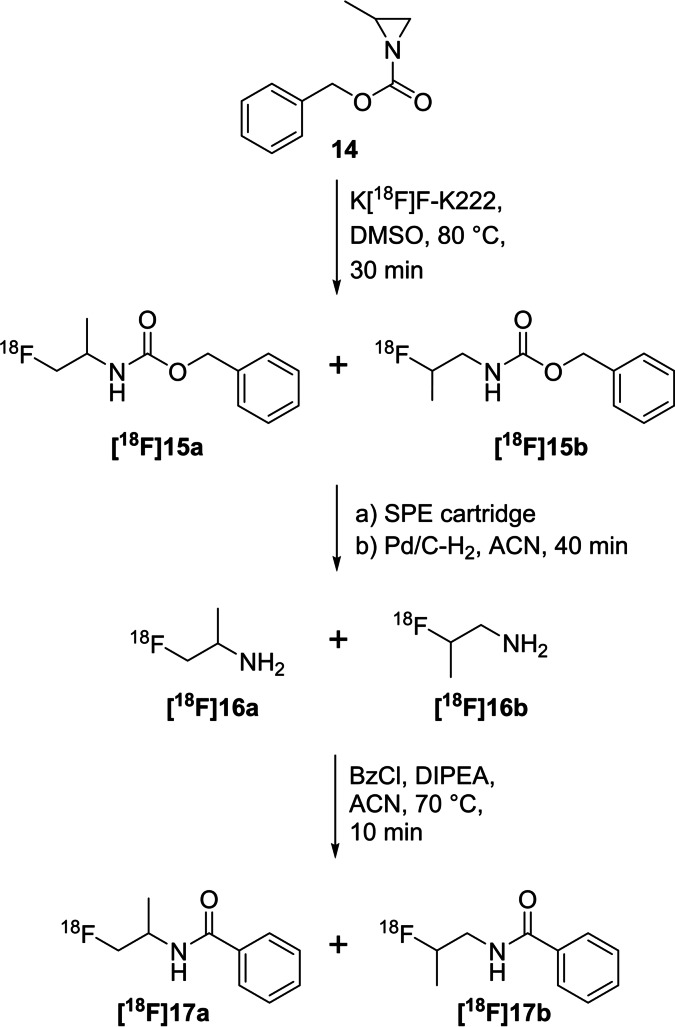
Radiofluorination of *N‐*Cbz‐2‐methylaziridine **14** including purification, deprotection and reaction with benzyl chloride to yield **[^18^F]17 a** and **[^18^F]17 b**.

A radiolabelling method for the generation of ^18^F‐labelled amine intermediates was developed using benzyloxycarbonyl (Cbz)‐protected 2‐methylaziridine **14** and [^18^F]fluoride. The ring opening procedure yielded two regioisomeric products due to the unsymmetrically substituted aziridine ring.[^48^, ^49^] The Cbz‐protected precursor **14** was prepared from benzyl chloroformate and 2‐methylaziridine. The radiolabelling was performed using K[^18^F]F‐K222 in DMSO at 80 °C for 30 min, showing 40 % to 80 % radiochemical conversion (RCC) as summarised in Scheme 6.

Both Cbz‐protected regioisomers **[^18^F]15 a**,**b** were subsequently purified using an SPE (solid‐phase extraction) cartridge prior to the quantitative deprotection with Pd/C‐H_2_ to yield **[^18^F]16 a**,**b**. In a proof‐of‐concept study, both radiofluorinated amines **[^18^F]16 a**,**b** were reacted with benzyl chloride in the presence of DIPEA (*N,N*‐diisopropylethylamine) as base, yielding the final radiotracers **[^18^F]17 a**,**b** with 65 % RCY and a ratio of 85 : 15 (**[^18^F]17 a:[^18^F]17 b**) of both regioisomers verified by HPLC. The identification was carried out with the respective nonradioactive compounds.

Generally, the nucleophilic attack of [^18^F]fluoride is possible at both carbon atoms (C‐2 and C‐3) of the three‐membered aziridine ring. Due to the slight steric hinderance of the methyl group at the C‐2 carbon atom of **14**, the formation of 1‐[^18^F]fluoro‐2‐propanamine (**[^18^F]16 a**) as the major product was favoured (ratio 85 : 15). 2‐[^18^F]Fluoro‐1‐propanamine (**[^18^F]16 b**) was obtained as the minor product after catalytic hydrogenation.

**Scheme 7:**
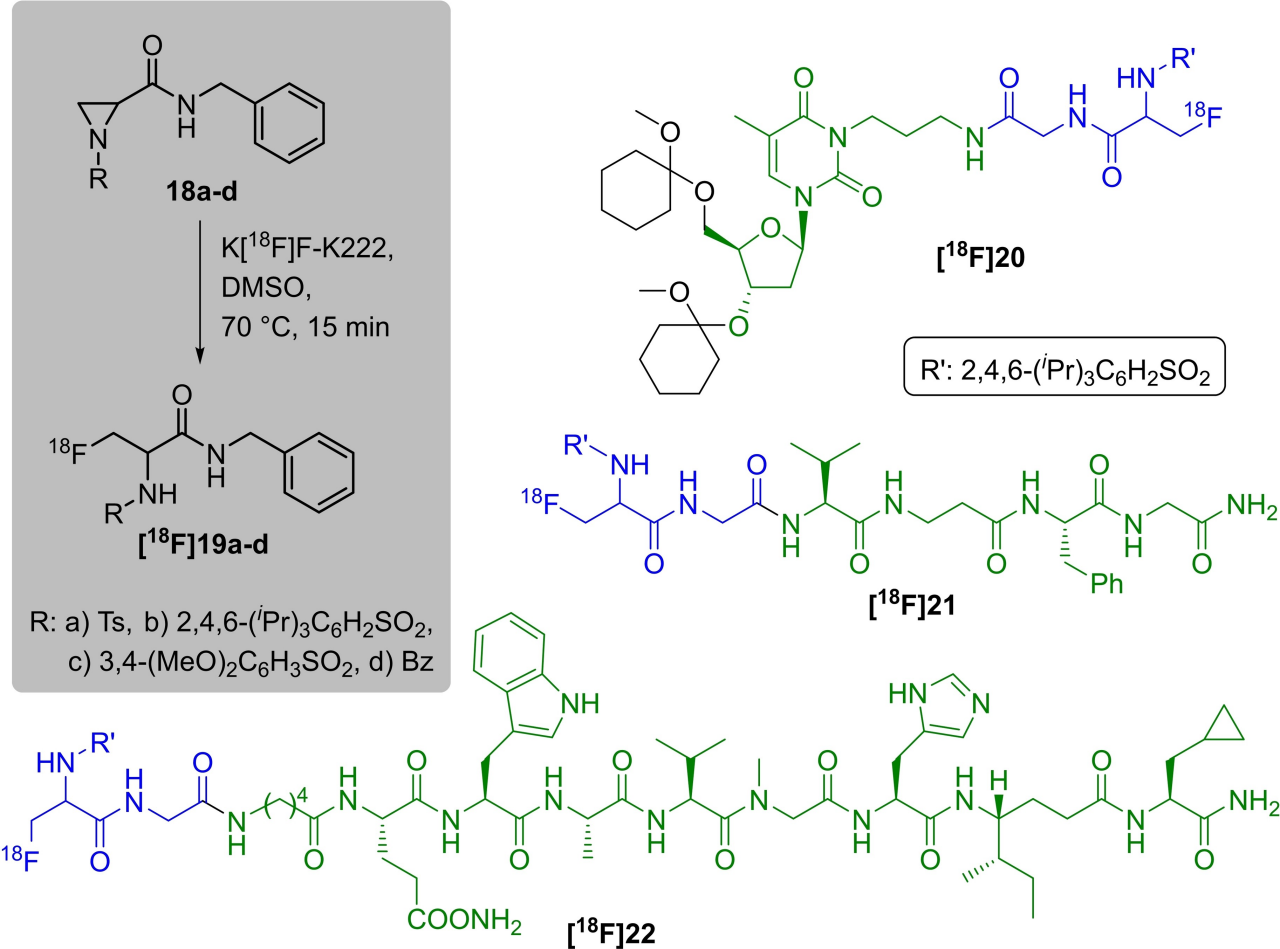
General radiolabelling procedure to prepare α‐amino‐β‐[^18^F]fluoropropanamides **[^18^F]19 a**–**d** (grey box) and selected ^18^F‐labelled biomolecules **[^18^F]20‐[^18^F]22** using this method.

Unsymmetrically functionalised aziridine‐2‐carboxamides are the basis for the direct nucleophilic introduction of fluorine‐18 in a regioselective manner, yielding α‐amino‐β‐[^18^F]fluoropropanamide derivatives (Scheme 7).^[50]^ Four model compounds **18 a**–**d** were chosen to point out the labelling conditions and the influence of the functional group connected to the nitrogen atom of the aziridine. Electron‐withdrawing sulfonyl groups have been found superior for the fluorine introduction at elevated temperatures in contrast to the benzoyl group. The radiofluorination experiments were carried out with 2 mg of the respective precursor in DMSO and K_2_CO_3_ or Cs_2_CO_3_ as base at 70 °C for 15 min, showing up to 97 % conversion for the model compounds **[^18^F]19 a**–**c**. Benzoyl derivative **[^18^F]19 d** was not formed. The preparation of the nonradioactive reference compounds was performed with KF‐ K222 in DMSO at 50 °C for 1 h. A formation of the respective regioisomer was not observed, but small amounts of hydrolysed or rearranged by‐products were identified. Based on these findings, three aziridine‐functionalised biomolecules were prepared and directly radiolabelled with [^18^F]fluoride under the above‐elaborated conditions. Notably, both peptides were radiolabelled without protection of the amino acid side chains. The shorter peptide, **[^18^F]21**, was obtained in 16 %, the longer peptide **[^18^F]22** in 7 % and the thymidine derivative **[^18^F]20** in 87 % (all values represent radiochemical conversions quantified by analytical HPLC).

**Scheme 8:**
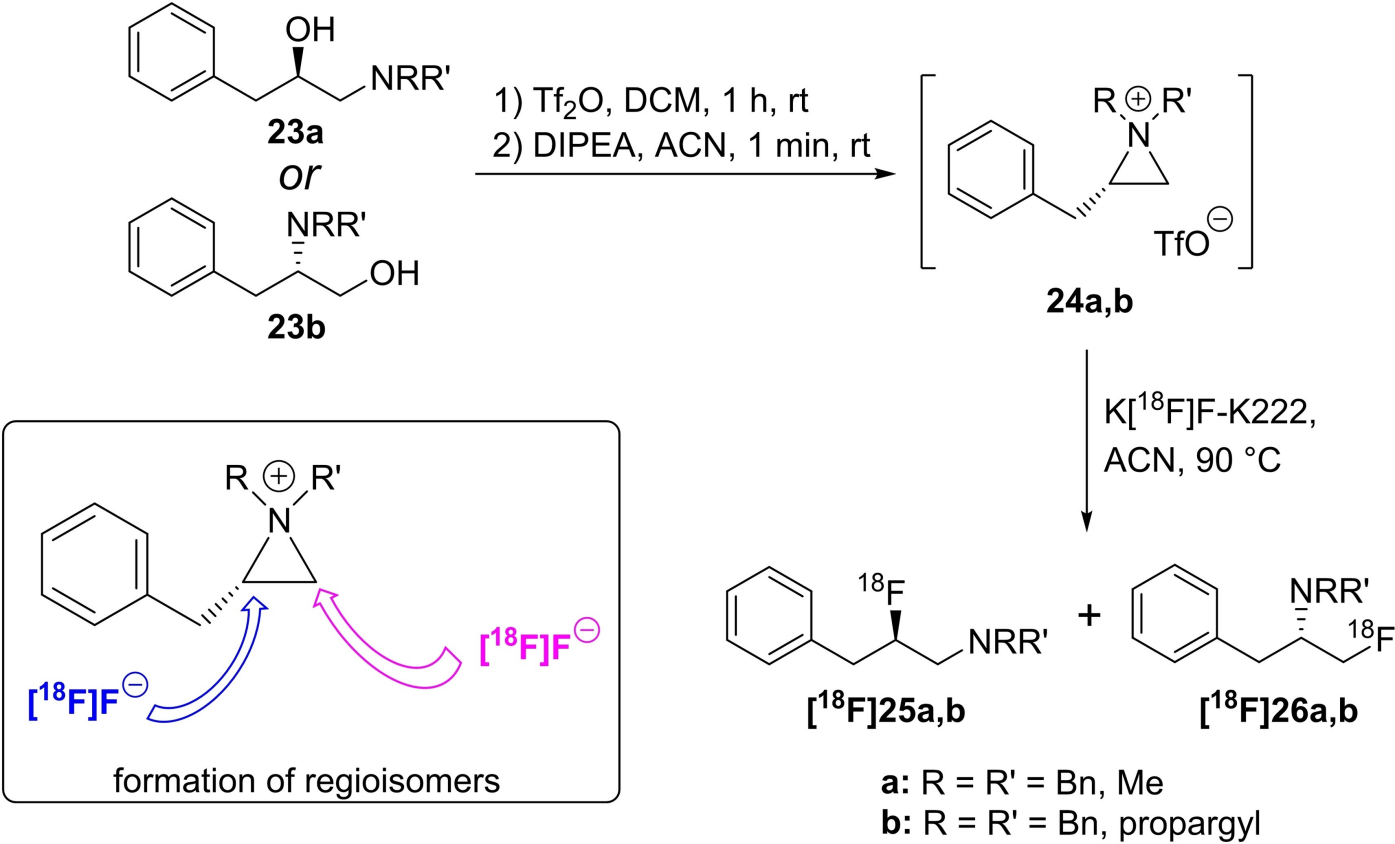
One‐pot radiofluorination of aminoalcohols **23 a** or **23 b** via intermediate cyclisation to the aziridinium salts **24 a**,**b** followed by treatment with [^18^F]fluoride to yield regioisomers **[^18^F]25 a**+**[^18^F]26 a** and **[^18^F]25 b**+**[^18^F]26 b**.

Médoc and Sobrio prepared compounds with a 2‐[^18^F]fluoroethylamine pattern from the appropriate precursors with a 2‐hydroxyethyl moiety, relying on the anchimeric assistance of the neighbouring amine.^[51]^ For this purpose, compounds **23 a**,**b** were used to form the aziridinium intermediates **24 a**,**b**. The subsequent nucleophilic ring opening of the aziridinium intermediate by [^18^F]fluoride at room temperature led to the formation of both radiofluorinated regioisomers **[^18^F]25 a** and **[^18^F]26 a** as well as to **[^18^F]25 b** and **[^18^F]26 b** with RCYs of up to 77 %. To prepare the aziridinium precursors **24 a**,**b** by forcing the ring closure, the OH groups of **23 a**,**b** were sulfonylated using triflic anhydride with DIPEA as base to achieve the highest yields. Other reagents like methanesulfonic anhydride and other bases gave lower yields. Afterwards, different radiolabelling conditions were tested with different bases and crown ethers. Based on these results, a one‐pot procedure, including the ring closure and subsequent nucleophilic attack of [^18^F]fluoride (Scheme 8), was developed. In principal, the nucleophilic attack is possible at both carbon atoms of the aziridinium ring (see box in Scheme 8). Thus, both regioisomers **[^18^F]25 a/[^18^F]26 a** and **[^18^F]25 b/[^18^F]26 b** were obtained independently of the (radio‐)fluorination method, but with a tendency to form the isomer with the lesser steric demand.

**Scheme 9:**
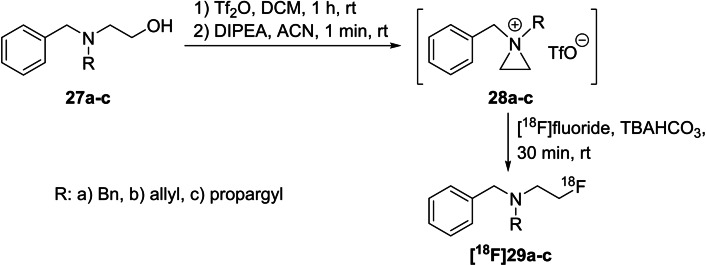
One‐pot radiofluorination of β‐aminoalcohols **27 a**–**c** via intermediate cyclisation to aziridinium salts **28 a**–**c** followed by treatment with [^18^F]fluoride to obtain **[^18^F]29 a**–**c**.

To expand the scope, this method was extended to a larger number of compounds and was applied to the radiofluorination of aziridinium salts obtained from β‐hydroxyethylamines to yield *N,N*‐disubstituted 2‐[^18^F]fluoroethylamines (Scheme 9). Due to the symmetric unsubstituted aziridinium ring, the ring opening led to a single radiofluorinated product. Three model compounds **27 a**–**c** were treated with [^18^F]fluoride using the labelling methods described above to obtain the desired products **[^18^F]29 a**–**c** in RCYs of 14 % to 31 %.

The respective nonradioactive reference compounds were prepared using the same precursors **27 a**–**c**, which were first converted into the aziridinium derivatives **28 a**–**c** with trifluoromethanesulfonic anhydride and DIPEA as base. Next, the cyclic intermediates were treated with TBAF to obtain the desired fluoro compounds after chromatographic purification.

**Scheme 10:**
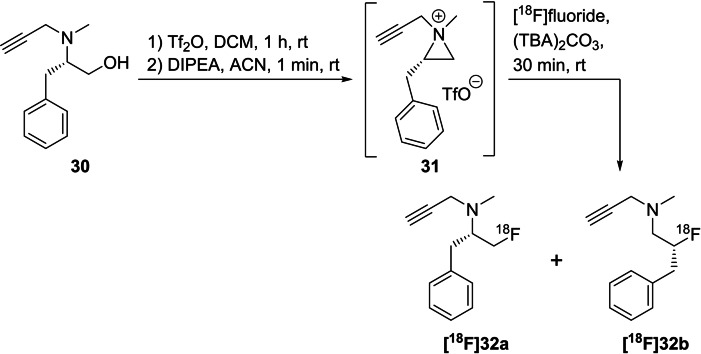
One‐pot radiofluorination of aminoalcohol **30** via intermediate cyclisation to aziridinium salts **31** followed by treatment with [^18^F]fluoride and the influence of regioisomer formation.

In 2015, the same group expanded their portfolio to label various *N,N*‐disubstituted‐β‐aminoalcohols (Scheme 10) using the one‐pot method described above.^[52]^ They investigated the influence of the temperature on the radiochemical yield and on the ratio of the formed regioisomers and found that radiochemical yields varied depending on the substituent connected to the nitrogen atom. In most cases and as expected, the RCY increased with higher reaction temperature. However, when propargyl was used as a substituent, exemplarily shown with precursor **30**, a degradation of the 2‐fluoropropan‐1‐amine isomer **[^18^F]32 b** occurred at 90 °C, leading to a regiospecific reaction to [^18^F]fluorodeprenyl **[^18^F]32 a** in the radiolabelling of **30**. In contrast, a ratio of 55 : 45 for both tracers **[^18^F]32 a** : **[^18^F]32 b** was found for radiolabelling of **30** at room temperature.

The stability of the product depends on the substituents, and the following order was established for the radiolabelling procedure at 90 °C: *N,N*‐dimethylamine=*N,N*‐methylpropargylamine>*N,N*‐methylbenzylamine≫piperidyl=*N,N*‐dipropargylamine=*N,N*‐diallylamine≫*N,N*‐dibenzylamine.

**Scheme 11:**
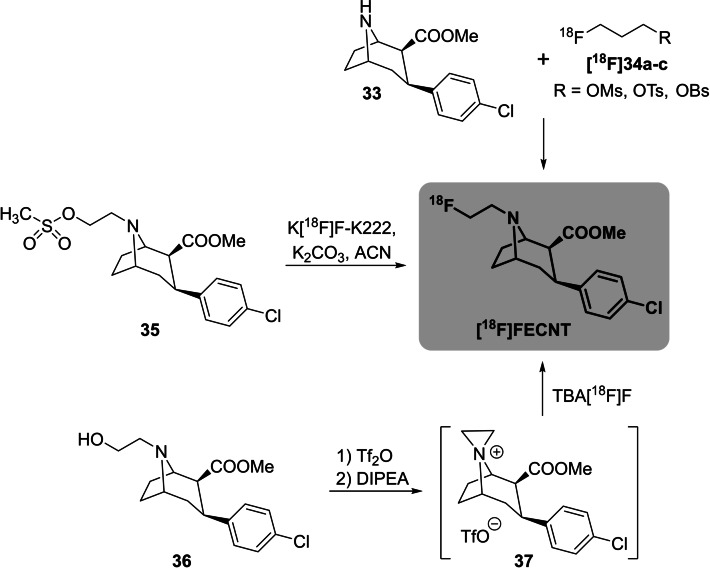
Three different radiofluorination routes for the preparation of **[^18^F]FECNT**.

Furthermore, this method was applied to the preparation of **[^18^F]FECNT**, a radiopharmaceutical used to image dopamine transporters in the brain.[^53^, ^54^] The alkylation of the nortropane precursor **33** using different 2‐[^18^F]fluoroethylsulfonates **[^18^F]34 a**–**c**[^55^, ^56^, ^57^] or bromide is conducted in a two‐step procedure with overall RCYs ranging from 16.5 % to 40 % (d.c.) or, alternatively, in one step from the respective mesylate precursor **35**,[^58^, ^59^] but this precursor **35** has been described to be unstable. As an alternative, this working group reported a one‐pot procedure starting from the respective aminoalcohol **36**, which was first transferred into the aziridinium triflate intermediate **37** and then subsequently radiolabelled using the above‐mentioned conditions to obtain **[^18^F]FECNT** in a RCY of 12 % (rt) or 21 % (90 °C). All three routes are detailed in Scheme 11.

Methyl aziridine‐2‐carboxylate was equipped with five different electron withdrawing protecting groups (Ts, Ns, Boc, Fmoc, Cbz) for the preparation of α‐[^18^F]fluoro‐β‐alanine or β‐[^18^F]fluoroalanine.^[60]^ First of all, the ^18^F‐introduction by ring opening with [^18^F]fluoride was only successful for derivatives containing Boc, Cbz or Ts as activating group (exemplarily shown for precursor **38**). For these precursors, labelling conditions were optimised by varying the solvent, temperature (including microwave conditions), precursor amount, base, and time. Additionally, an HPLC procedure was established to separate both regioisomers. The highest ^18^F‐incorporation with a RCY of 45 % was found using temperatures ≥100 °C combined with microwave support and TEAHCO_3_ as mild base, furthermore using DMSO as solvent. Notably, the methyl ester of **[^18^F]39 a**,**b** was partially cleaved during the labelling procedure, whereas the activating group was only cleaved after labelling under strong acidic conditions together with the remaining ester to yield the desired radiotracers **[^18^F]40 a**,**b**.

**Scheme 12:**
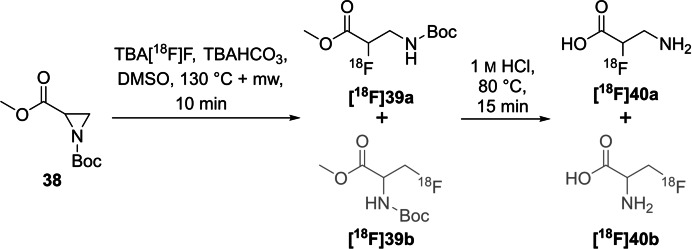
Labelling procedure for the preparation of α‐[^18^F]fluoro‐β‐alanine **[^18^F]40 a** in two steps, including ring opening by nucleophilic attack followed by acidic deprotection. β‐[^18^F]fluoroalanine **[^18^F]40 b** (grey structure) was not obtained.

α‐[^18^F]Fluoro‐β‐alanine **[^18^F]40 a** was exclusively obtained after the ring‐opening for labelling of the Boc‐ and Cbz‐activated aziridines and after ester hydrolysis and activation group removal (Scheme 12). The aziridine was attacked by [^18^F]fluoride exclusively at the most substituted α‐carbon atom. No other regioisomer was detected. Interestingly, reports on ring opening using the nonactivated isopropyl aziridine‐2‐carboxylate, for example with HF/pyridine, showed completely opposite regioselectivity with attack at the unsubstituted β‐carbon atom.[^61^, ^62^]

**Scheme 13:**
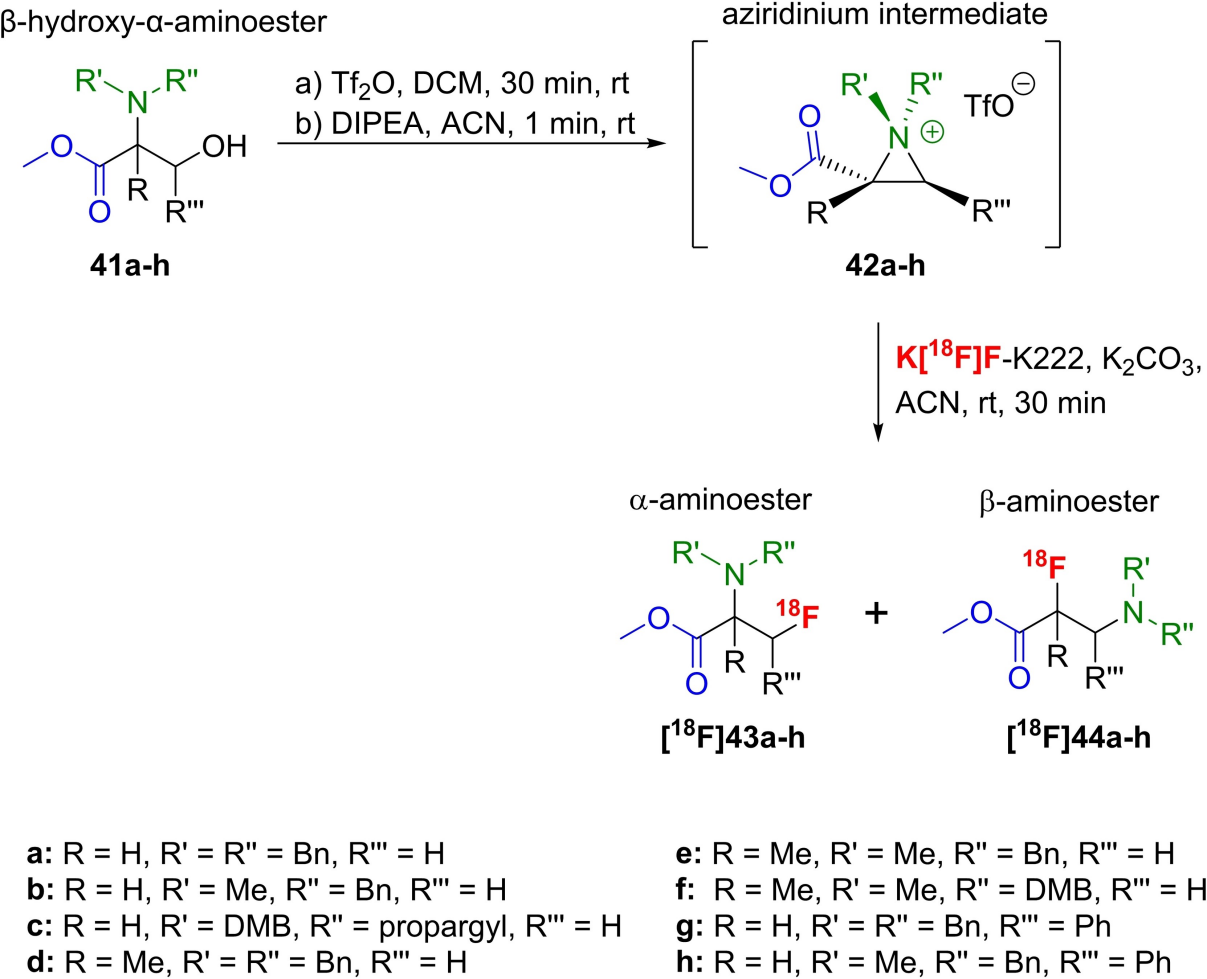
2‐step one‐pot radiofluorination procedure for the preparation of serine‐based fluorine‐18‐containing α‐ and β‐amino acids **[^18^F]43 a**–**h** and **[^18^F]44 a**–**h**.

Another convenient access to [^18^F]fluoroaminoesters under mild conditions by deoxyradiofluorination of β‐hydroxy‐α‐aminoesters derived from serine, α‐methyl‐serine, or β‐phenyl‐serine was described in 2019.[^63^, ^64^] For this purpose, a three‐step procedure was developed, starting from differently *N*,*N*‐alkylated compounds **41 a**–**h** (R’ and R’’=Bn, DMB, Me, propargyl), which were prepared and first treated with triflic anhydride in dichloromethane for 1 h at rt. Next, DIPEA in acetonitrile was added as base to generate the respective aziridinium precursor intermediates **42 a**–**h**. Afterwards, K[^18^F]F‐K222 and K_2_CO_3_ as base in acetonitrile were added and the final mixture was stirred at rt for 30 min. This subsequent radiofluorination, under optimised conditions, yielded the desired ^18^F‐radiotracers **[^18^F]43 a**–**h** and **[^18^F]44 a**–**h** as isomeric mixture in different ratios and in RCYs ranging from 10 % to 75 % (Scheme 13).

In principle, two regioisomers are possible from radiofluorination by nucleophilic attack of the [^18^F]fluoride due to the two differently substituted carbon atoms of the three‐membered ring. Notably, both isomers were found when using phenylserine **41 g**,**h** (R=H and R′′′=Ph) with the α‐isomer **[^18^F]43 g**,**h** dominating. In contrast, the radiofluorination is completely regioselective when using serine or methylserine derivatives **41 a**,**b**,**d**,**e** (R=H or Me and R′′′=H). Only the respective β‐aminoesters **[^18^F]44 a**,**b**,**d**,**e** were obtained. No radiolabelled product was found when using the dimethoxybenzyl group (R′=DMB) for amine protection as found in precursors **41 c**,**f**, probably due to the steric hinderance of this bulky group.

The appropriate reference compounds were prepared using either the direct deoxyfluorination with DAST or the procedure with the aziridinium precursors and TBAF as fluorine source, also yielding both regioisomers for R=H and R′′′=Ph and, in the case of R=H and R′′′=H, Me, exclusively the β‐isomer independently of the (radio)fluorination method.

## 3 Azetidinium Salts

**Scheme 14:**
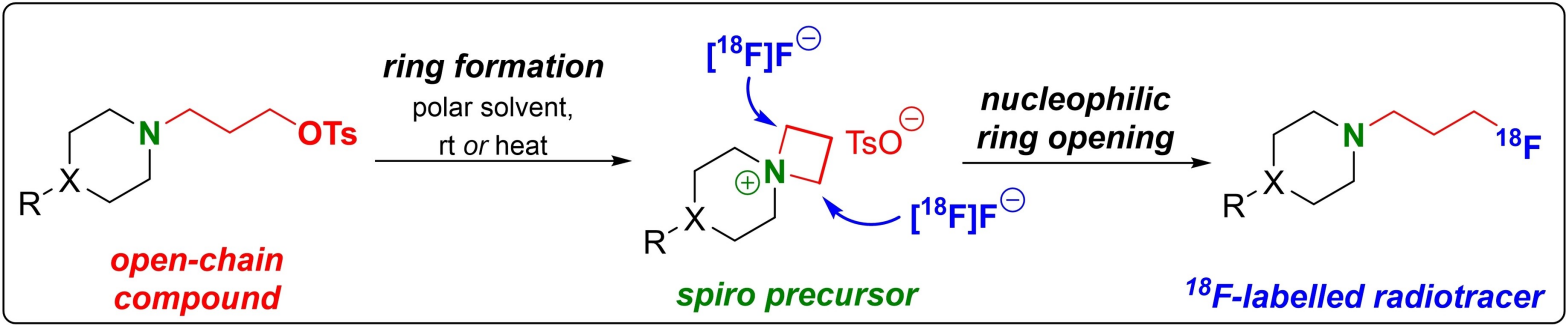
Overview about the mild radiofluorination of azetidinium salts by nucleophilic ring‐opening.

Azetidinium salts are organic four‐membered heterocyclic compounds with one positively‐charged nitrogen atom in the ring. They are sensitive to nucleophilic attacks at ring positions C‐2 and C‐4 due to the positive charge of the nitrogen atom that leads to a s_n_ 2 reaction yielding the respective ring‐opened product.^[65]^ Of all small cyclic compounds, the four‐membered representatives are generally the most difficult to synthesise. However, a number of new synthetic methods have been elaborated,[^66^, ^67^, ^68^, ^69^] including the enantioselective nucleophilic ring opening of these salts by fluoride, leading to γ‐fluoropropylamines (Scheme 14).^[70]^

**Scheme 15:**
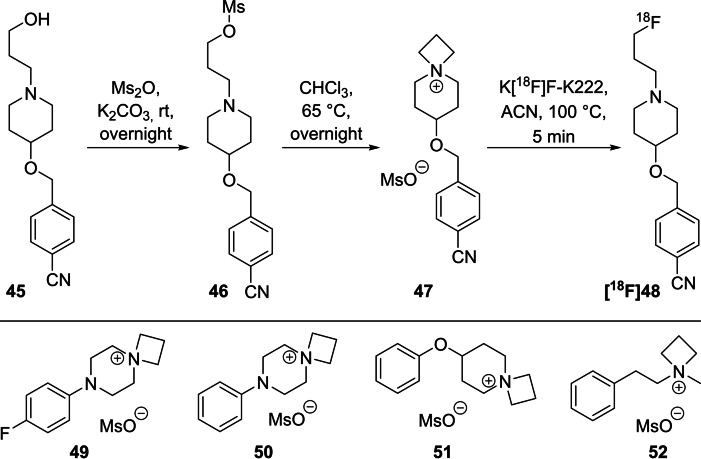
Preparation of *spiro* azetidinium precursor **47**, radiofluorination to **[^18^F]48** and structure of *spiro* precursors **49**–**52**.

The first investigation to introduce fluorine‐18 via azetidinium precursors was reported in 2004 by Kiesewetter and Eckelman.^[71]^ They aimed to prepare 4‐{[1‐(3‐[^18^F]fluoropropyl)‐4‐piperidinyl]‐methoxy}benzonitrile (**[^18^F]48**), a ^18^F‐radiotracer to image the sigma1 receptor with high selectivity.^[72]^ During the synthesis of the precursor **47**, they found that the open‐chain mesylate derivative **46** spontaneously cyclised, forming the respective azetidinium salt. In‐depth NMR investigations were carried out to elucidate the structure of the azetidinium salt. The following radiolabelling reaction was performed in acetonitrile, using K_2_CO_3_ as base and the azeotropically dried K[^18^F]F‐K222‐complex at a minimum temperature of 80 °C to maximise the RCY, obtaining **[^18^F]48** in a very short reaction time of 5 min (Scheme 15). To get a deeper understanding of this reaction, four additional azetidinium compounds **49**–**52** with mesylate as ion were prepared and radiolabelled with RCYs ranging between 60 % and 72 %. Interestingly, no difference in RCY was found at high temperatures (>80 °C) between the open‐chain precursor **46** and the azetidinium precursor **47**, probably due to the intermediate ring closure, whereas a huge difference occurs at 41 °C. The azetidinium precursor **47** showed a high conversion of 26 % in comparison to the open‐chain precursor **46** with only 1.4 %, demonstrating a high and preferred ^18^F‐incorporation into the azetidinium salts even at rt or slightly elevated temperatures.

Interestingly, the respective nonradioactive reference compounds were not prepared from the *spiro* precursors, instead 1‐bromo‐3‐fluoropropane and the appropriate secondary amines have been used for this purpose.

**Scheme 16:**
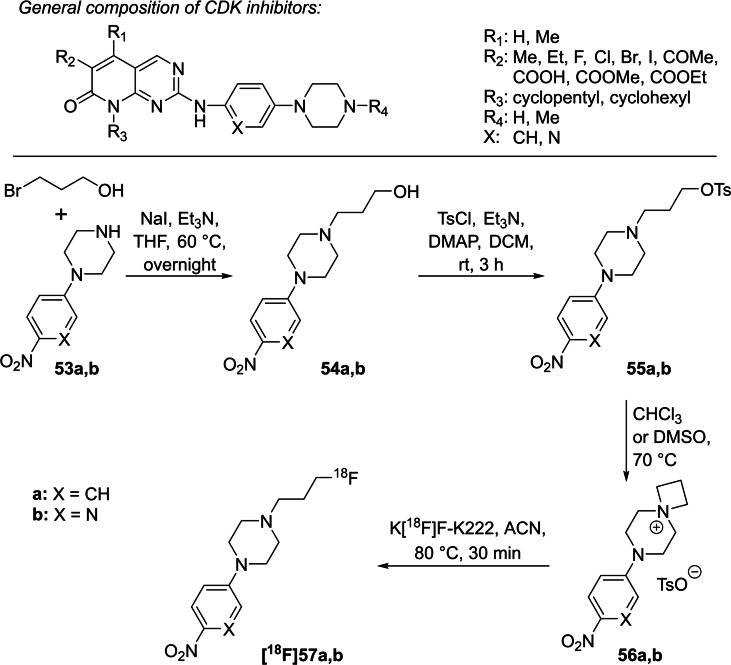
Gereral composition of CDK inhibitors and preparation of two model radiotracers **[^18^F]57 a**,**b**, which act as intermediates for CDK inhibitors.

In 2011, Mamat et al. used 1‐(3‐[^18^F]fluoropropyl)piperazines as model compounds to prepare ^18^F‐radiotracers based on inhibitors to image cyclin‐dependent kinases (CDK),^[73]^ since the piperazine moiety is a basic structural element of these CDK‐inhibitors (Scheme 16).^[74]^ They started with 1‐(4‐nitrophenyl)piperazine **53 a** and 1‐(6‐nitropyridin‐3‐yl)piperazine **53 b**, respectively, which were treated with 3‐bromopropanol to introduce the 3‐hydroxypropyl chain. After tosylation of the alcohol functions of **54 a**,**b**, the open‐chain derivatives **55 a**,**b** were obtained, which tend to cyclise spontaneously to the *spiro* salts **56 a**,**b** even at room temperature and much better in polar solvents like DMSO. Both azetidinium precursors **56 a**,**b** were then treated with the azeotropically dried K[^18^F]F‐K222‐complex at 80 °C for 30 min, yielding both ^18^F‐compounds **[^18^F]57 a**,**b** in RCYs >88 %. Interestingly, a high RCC of >65 % was observed for both *spiro* precursors even after 5 min at 80 °C.

**Scheme 17:**
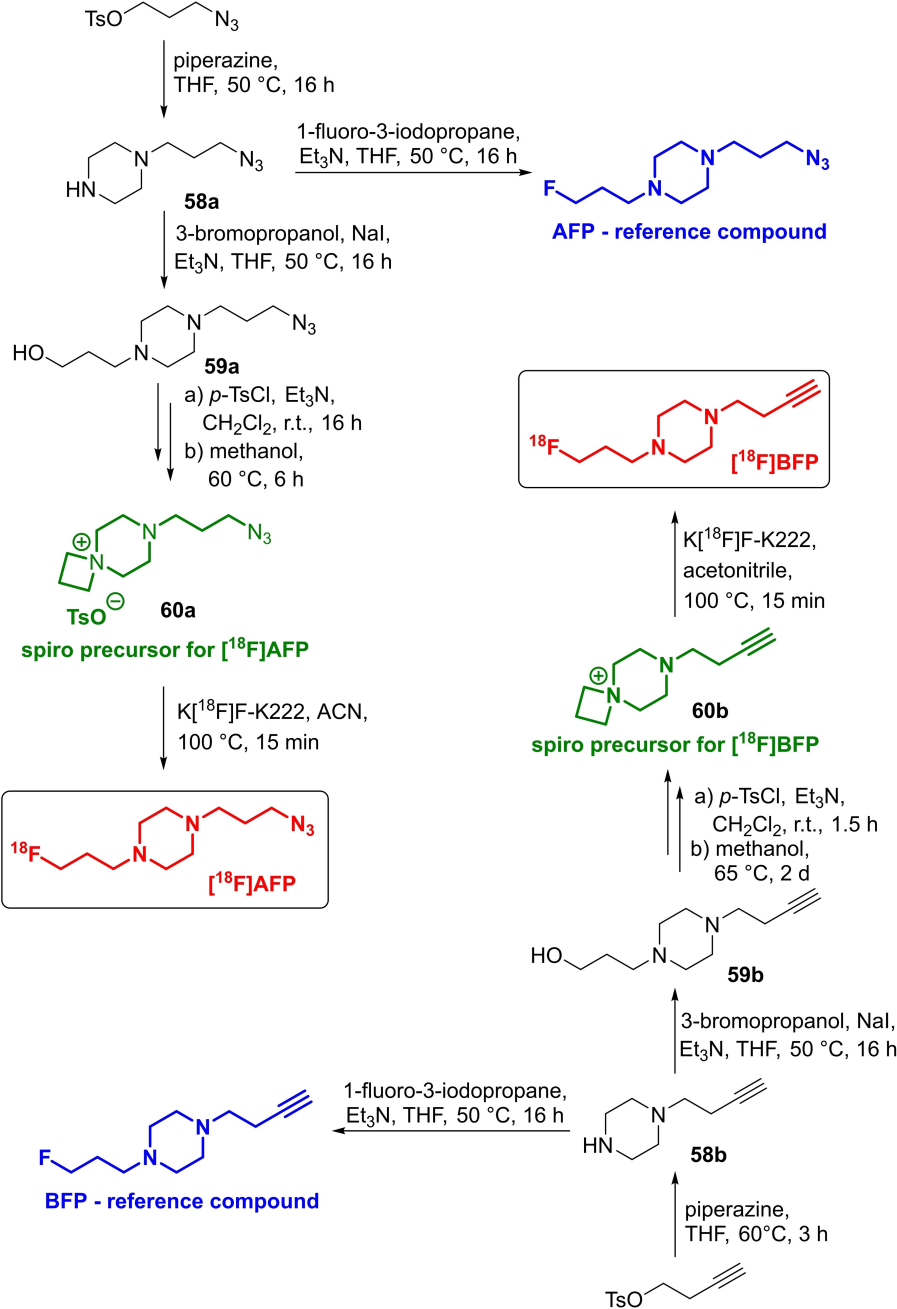
Preparation of the both *spiro* precursors **60 a**,**b** (green), the reference compounds **AFP** and **BFP** (blue) as well as both radiolabelling building blocks **[^18^F]AFP** and **[^18^F]BFP** (red).

In 2013, this method was transferred, by the same group, to prepare two radiolabelling building blocks **[^18^F]AFP** and **[^18^F]BFP** for bioorthogonal ^18^F‐introduction,^[75]^ which can be used for labelling purposes either with the traceless Staudinger Ligation^[76]^ or using Cu‐mediated or ‐free click reactions.[^77^, ^78^] Based on the piperazine skeleton, the respective reference compounds **AFP** and **BFP** as well as the *spiro* precursors **60 a**,**b** were synthesised by first introducing the azide or alkyne function into the piperazine moiety yielding **58 a**,**b** (Scheme 17). The next step involved the alkylation with 3‐bromopropanol to alcohols **59 a**,**b**, followed by subsequent tosylation with tosyl chloride. Both open‐chain tosylates were subsequently purified by means of column chromatography and then heated in methanol to induce cyclisation to *spiro* precursors **60 a**,**b** (43 % yield for both).

**Figure 1:**
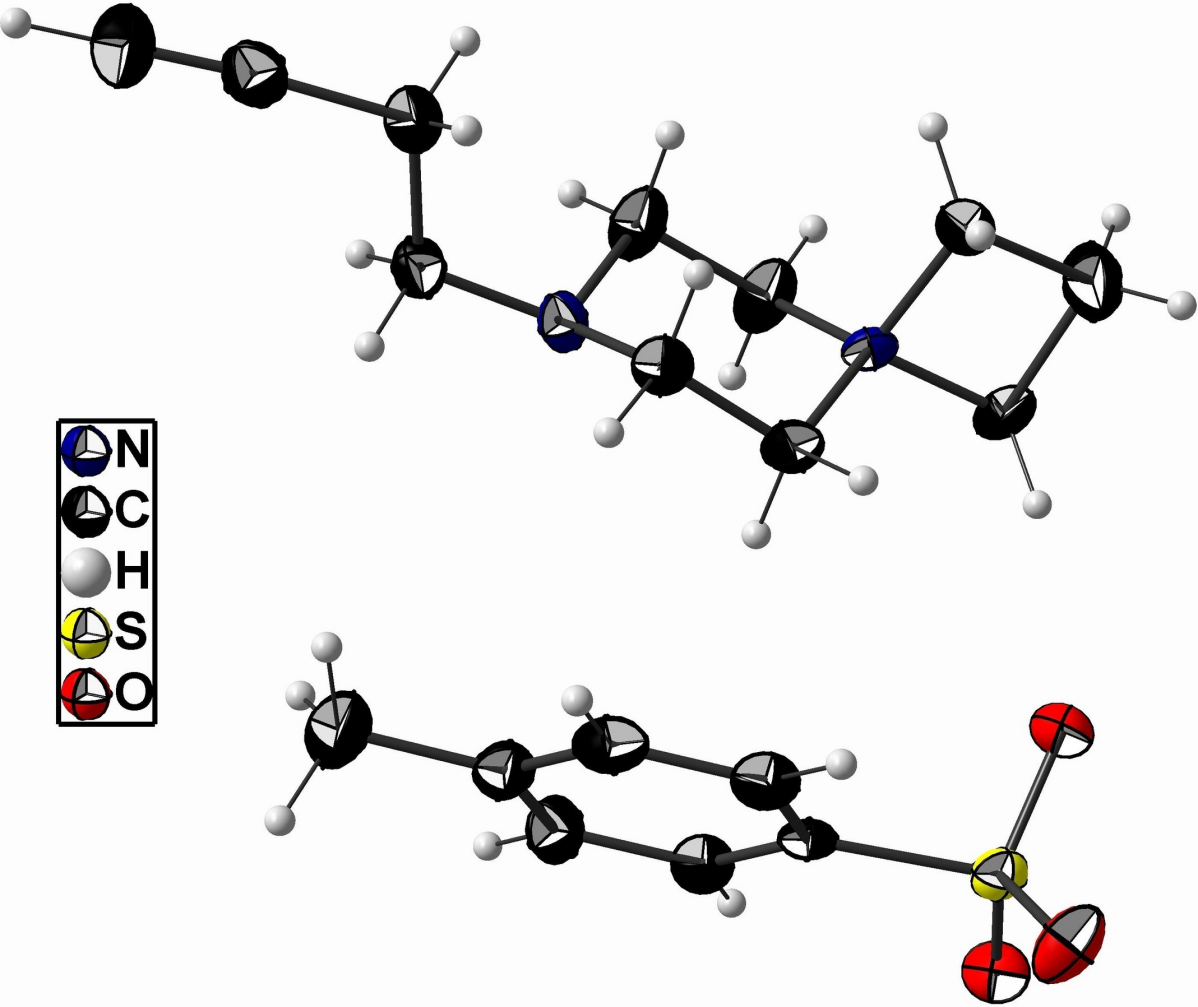
Molecular structure of the BFP precursor **60 b** in the crystal (ORTEP, displacement ellipsoids at the 50 % probability level).^[79]^

NMR analyses, MS and XRD confirmed the molecular structure of the formed *spiro* salts **60 a**,**b**. As one example, XRD analysis elucidated and confirmed the conformation of the two rings connected by the central nitrogen atom of BFP precursor **60 b** with a tosylate counterion, showing a chair conformation of the 6‐membered ring as shown in Figure 1.^[79]^

**Figure 2:**
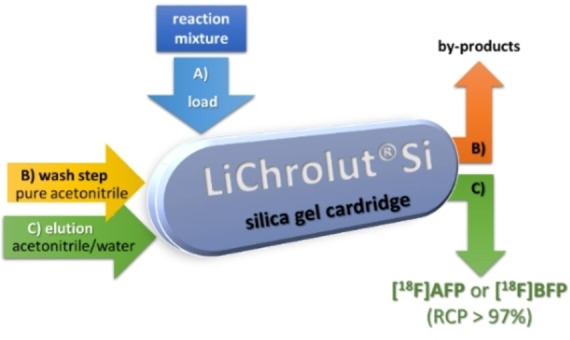
Elution scheme for the purification of **[^18^F]AFP** and **[^18^F]BFP** using a convenient silica gel cartridge.

The subsequent radiolabelling was accomplished with the *spiro* precursors **60 a**,**b**, which were treated with K[^18^F]F‐K222 and K_2_CO_3_ as base in acetonitrile at 100 °C for 15 min with nearly quantitative conversion of [^18^F]fluoride (start activity 1–12 GBq). Advantageously, a simple silica gel cartridge could be used for purification (Figure 2) of both radiolabelling building blocks **[^18^F]AFP** and **[^18^F]BFP**, leading to RCPs >97 %. Finally, an automated module synthesis was developed, yielding both building blocks in approx. 30 % RCY.[^80^, ^81^]

**Scheme 18:**
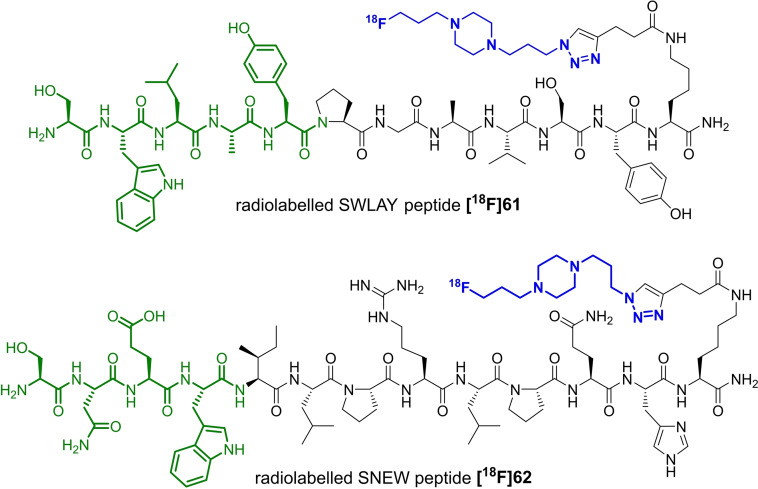
SWLAY peptide **[^18^F]61** and SNEW peptide **[^18^F]62** with click building block (blue) and main binding sequence (green).

In a proof‐of‐concept study, different azide‐functionalised amino acids were radiolabelled with these building blocks. Interestingly, it was possible to radiolabel small molecules like alkyne‐functionalised amino acids with **[^18^F]BFP** using Cu‐mediated click‐radiolabelling reactions. However, this building block was not found suitable with alkyne‐functionalised peptides^[81]^ due to the formation of bis‐alkynes via the Glaser coupling.^[82]^ Instead, **[^18^F]AFP** was used for the radiolabelling of the SNEW peptide **[^18^F]62**,^[83]^ which is known to be a substrate for the EphB4 receptor, and for radiolabelling the SWLAY peptide **[^18^F]61**,^[84]^ (Scheme 18) which is known to be a substrate for the EphA2 receptor.^[85]^

**Scheme 19:**
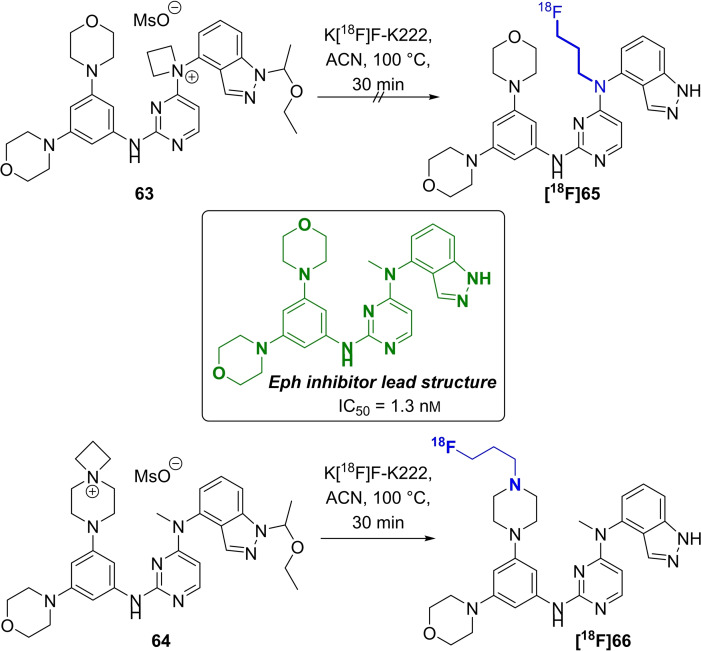
Structure of the original EphB4 inhibitor (box) and radiolabelling procedure for two azetidinium‐based precursors **63** and **64** to obtain **[^18^F]65** and **[^18^F]66**.

A high‐affinity EphB4 receptor ligand (box in Scheme 19) published by Bardelle et al.^[86]^ was used as basis for the development of potential PET radiotracers with fluorine‐18 and carbon‐11.[^87^, ^88^] The position for the introduction of carbon‐11 was easy to find because of the prominent methyl group in the molecule, allowing for the use of [^11^C]methyl iodide for radiolabelling.

In silico methods were used to investigate the suitability of different positions in the native molecule prior to the introduction of [^18^F]fluoride, aiming to prevent a loss of affinity to the EphB4 receptor. These studies resulted in the identification of two ^18^F‐radiotracers with [^18^F]fluoropropyl moieties (Scheme 19, highlighted in blue). Two *spiro*‐ammonium precursors **63** and **64** were prepared with an ethoxyethyl protecting group to avoid complications during the ^18^F‐radiolabelling. The introduction of [^18^F]fluoride followed standard conditions with acetonitrile as solvent with K[^18^F]F‐K222 and K_2_CO_3_ as base. The ^18^F‐radiotracer **[^18^F]66** was obtained in 34 % RCY after deprotection and purification using semipreparative HPLC, whereas the preparation of **[^18^F]65** was found to be impossible.

The PET‐radiotracer *N*‐3‐[^18^F]fluoropropyl‐2‐β‐carboxymethoxy‐3‐β‐(4‐iodophenyl)nortropane, also known as **[^18^F]FP‐CIT**, is a very prominent example for illustrating different strategies to obtain the [^18^F]fluoropropyl moiety. The cocaine analogue FP‐CIT^[89]^ has been described as a dopamine transporter ligand, and the radiolabelled compound **[^18^F]FP‐CIT**, together with **[^123^I]FP‐CIT**,^[90]^ are widely used for the detection or exclusion of nigrostriatal degeneration in patients with clinically uncertain Parkinsonian syndrome.[^91^, ^92^]

**Scheme 20:**
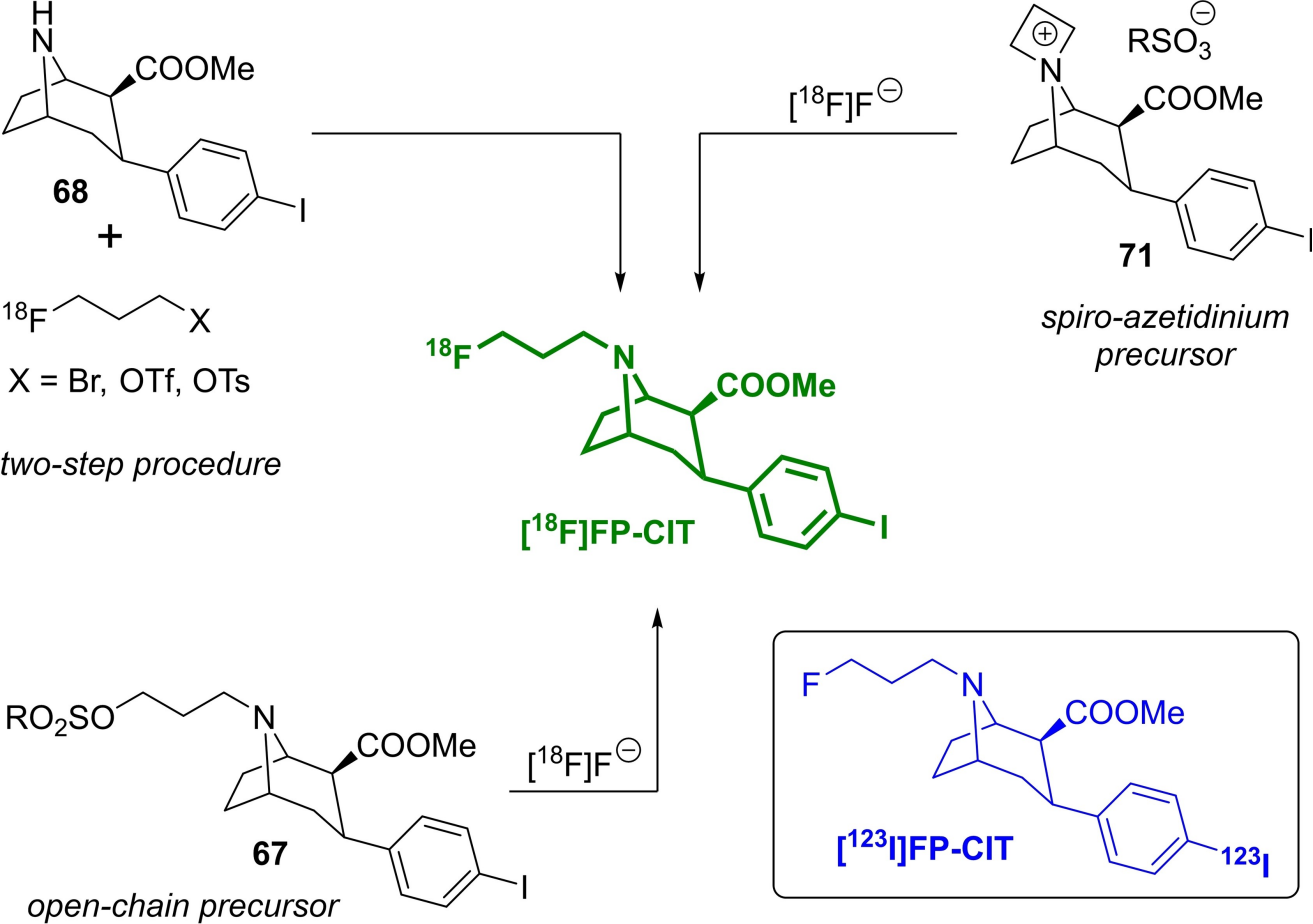
Structure of **[^18^F]FP‐CIT** (green), the radioiodinated analogue **[^123^I]FP‐CIT** (blue, in the box) and three radiolabelling methods to obtain **[^18^F]FP‐CIT** from the open‐chain precursor **67**, via a two‐step procedure with compound **68**, or using the *spiro*‐azetidinium precursor **71**.

Different radiofluorination methods using 3‐[^18^F]fluoropropyl tosylate (25.3±2.1 %),[^93^, ^94^] 3‐[^18^F]fluoropropyl triflate (RCY 70 %),^[95]^ or 3‐[^18^F]fluoropropylbromide (RCYs 2–49 %)[^19^, ^96^, ^97^] in a two‐step procedure with **68**, or the direct way either from the open‐chain precursor **67**[^28^, ^98^] or the azetidinium precursor **71** (RCY 92.4±3.6 %)^[99]^ have been described (Scheme 20), varying not only the solvent, the amount of precursor, the reaction time and the solvent.

**Scheme 21:**
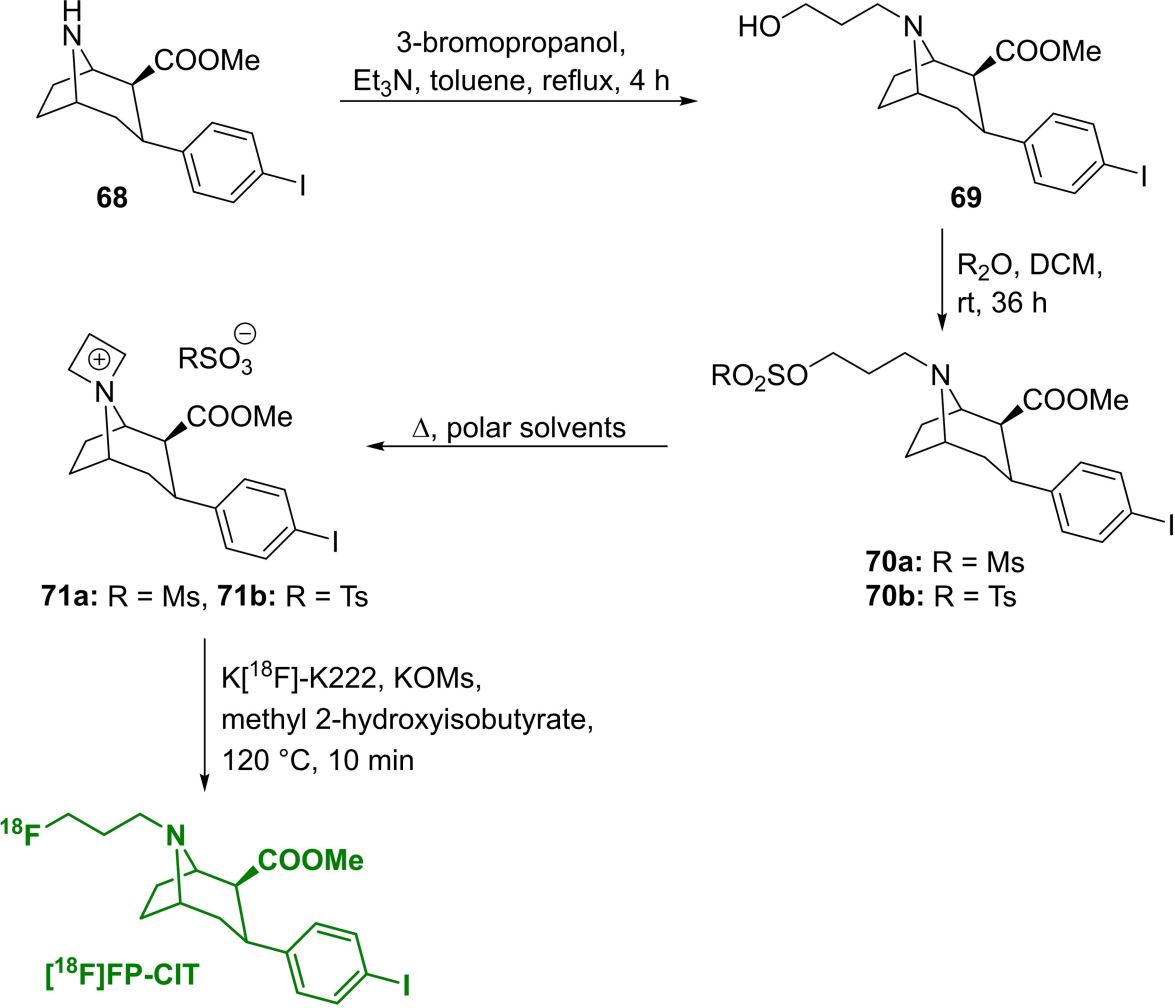
Synthesis of the *spiro* azetidinium precursors **70 a**,**b** and radiofluorination to obtain **[^18^F]FP‐CIT**.

The highest RCY of **[^18^F]FP‐CIT** was found when the respective azetidinium salt **71 a**,**b** was used as precursor, furthermore employing methyl 2‐hydroxyisobutyrate as solvent in a one‐pot reaction (Scheme 21). For this purpose, the precursor was prepared from cocaine skeleton **68** (also known as nor‐β‐CIT), which was treated with 3‐bromopropanol to yield **69** ^[100]^ and afterwards sulfonylated with Ts_2_O or Ms_2_O, respectively, to obtain the open‐chain precursors **70 a**,**b**.^[101]^ The cyclisation to the resulting precursors **71 a**,**b** was induced by heating in polar solvents. Interestingly, the ring opening was achieved after 1 h at 70 °C, using benzene as nonpolar solvent.

The fluorination was accomplished with TBAF in *t*‐BuOH at 80 °C. After 30 min, the nonradioactive reference compound FP‐CIT was obtained in a yield of 53 %. The respective radiolabelling was described for the open‐chain compound, leading to a decay‐corrected radiochemical yield of 35.8±5.2 % in an automated module synthesis.^[102]^ The respective mesyl *spiro* precursor **71 a** is commercially available.

The combination of using azetidinium salts as precursors and the minimalist approach for radiofluorinations was highlighted in a work of Omrane.^[103]^ This approach is based on the use of ammonium salts as precursors to avoid phase transfer catalysts and additives like Kryptofix K222 for the radiolabelling process with fluorine‐18.^[104]^ *spiro*‐Ammonium salts are therefore the ideal precursors. For this purpose, 7‐benzyl‐2‐hydroxy‐4‐azaspiro[3,5]nonan‐4‐ium *p*‐tosylate (**74**) was prepared according to a procedure published by Sladowska and co‐workers from **72** and **73**.^[105]^

**Scheme 22:**
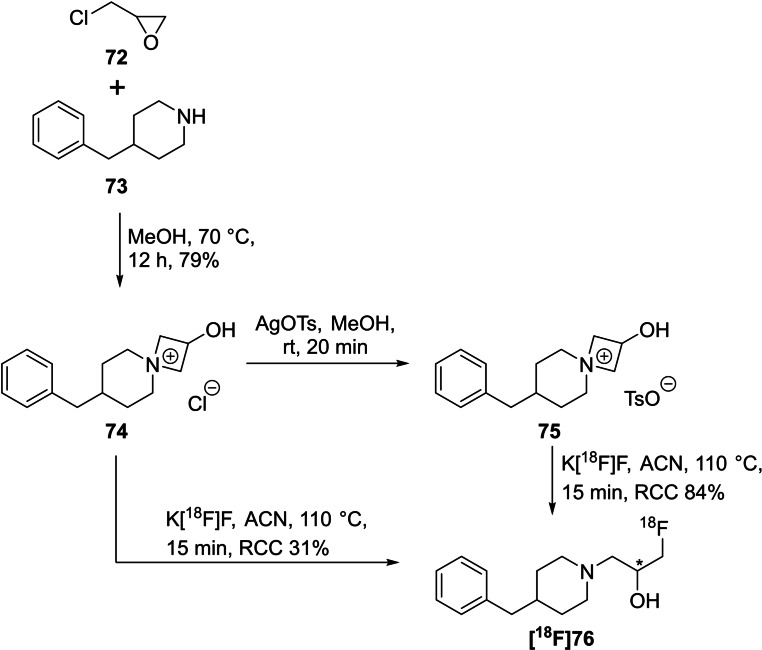
Proof‐of‐concept study for the radiosynthesis of 1‐(4‐benzylpiperidin‐1‐yl)‐3‐[^18^F]fluoropropan‐2‐ol (**[^18^F]76]**) from the *spiro* precursors **74** or **75** using the minimalist approach.

To perform the radiofluorination, [^18^F]fluoride was trapped on an anion‐exchange cartridge and eluted with the precursors **74** or **75**, respectively, dissolved in methanol. After changing the solvent to acetonitrile, the mixture was heated to 110 °C for 15 min. The initial experiments were performed with chloride as anion (precursor **74**); leading to a radioactive by‐product in high yields (38 %). After changing to tosylate as counterion (precursor **75**), the desired radiofluorinated compound **[^18^F]76** was obtained in up to 84 % when using acetonitrile as solvent at 110 °C for a reaction time of 15 min (Scheme 22). The respective reference compound was prepared in 51 % yield using TBAF(*t*‐BuOH)_4_ in acetonitrile at 75 °C after 4 h. Additionally, this method was transferred to the production of **[^18^F]AFP** from the respective *spiro* precursor **60 a**. A RCC of 94 % was observed under the above‐mentioned optimised conditions.

## 4 Conclusion

The use of aziridines, their aziridinium salts, and azetidinium salts represents a convenient access to obtain pharmacologically interesting compounds like β‐amino acids with either 2‐fluoroethylamino or 3‐fluoropropylamino moieties, respectively, as well as radiotracers with the corresponding 2‐[^18^F]fluoroethylamino or 3‐[^18^F]fluoropropylamino pattern, which were available mostly in high radiochemical yields and purities. In many cases, the respective precursors are readily accessible and the following radiofluorination proceeds smoothly under mild labelling conditions with high RCCs due to the anchimeric assistance of the nitrogen in β‐ or γ‐position. More stringent labelling conditions are required for the nucleophilic ^18^F‐introduction into aziridines. Furthermore, regioselectivity depends on sterically demanding functional groups. Additionally, methods and strategies were elaborated for a regioselective introduction of fluorine‐18 when using unsymmetrically substituted ring systems by tuning the reaction conditions and varying the substituents at the ring system.

## Conflict of interest

The authors declare no conflict of interest.

5

## Biographical Information

*Falco Reissig studied Chemical Engineering at the Hochschule für Technik und Wirtschaft Dresden earning his Master degree in 2017. In his PhD thesis, which he finished in 2021, he focussed on radiolabelling reactions especially working with alpha‐emitting radiometals. Since 2021, he works as PostDoc at the Institute of Radiopharmaceutical Cancer Research at the Helmholtz‐Zentrum Dresden‐Rossendorf*.

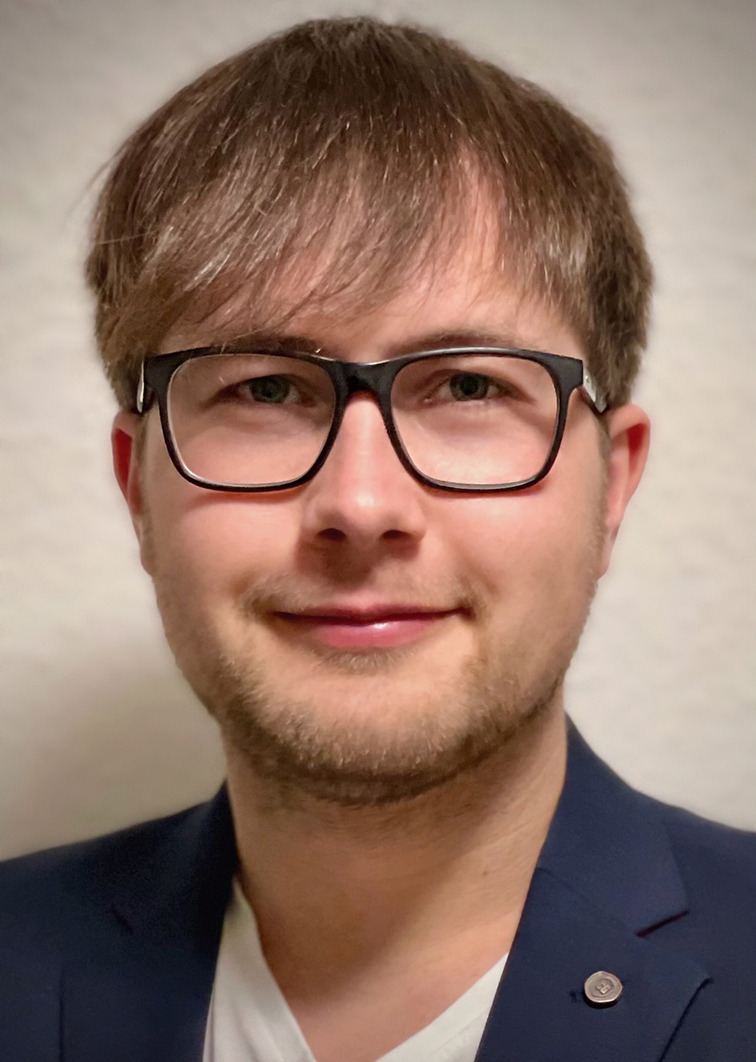

## Biographical Information

*Constantin Mamat studied Chemistry at the University of Rostock, where he completed his diploma in 2003 in the field of organosilicon chemistry and his PhD in 2007 working on carbohydrates. In 2016, he finished his habilitation at the Technical University Dresden. Since 2007, he works as a Senior Research Scientist at the Institute of Radiopharmaceutical Cancer Research at the Helmholtz‐Zentrum Dresden‐Rossendorf on the design of biorthogonal click reactions for fluorescence and radiolabelling and the preparation of alpha‐therapeutic agents*.

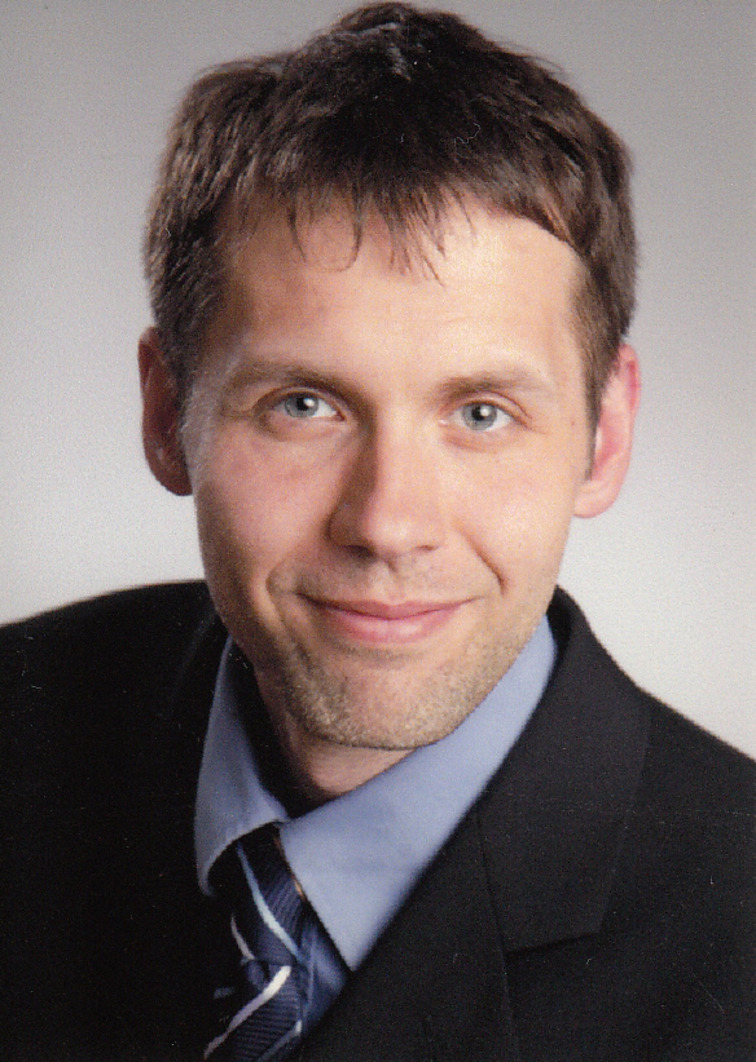

## Data Availability

Data sharing is not applicable to this article as no new data were created or analyzed in this study.
